# Development of an accurate and sensitive assay for 2-methoxyestradiol using derivatization and liquid chromatography-tandem mass spectrometry

**DOI:** 10.1016/j.plabm.2024.e00447

**Published:** 2025-01-09

**Authors:** Koji Takahashi, Masaki Takiwaki, Seketsu Fukuzawa, Yoshikuni Kikutani, Kentaro Abe, Tatsuya Higashi, Hironori Kobayashi

**Affiliations:** a Medical Equipment Business Operations, JEOL Ltd., Tokyo, Japan; b Faculty of Pharmaceutical Sciences, Tokyo University of Science, Chiba, Japan; c Laboratories Division, Shimane University Hospital, Shimane, Japan

**Keywords:** LC-MS/MS, 2-Methoxyestradiol, MPDNP-F, Estrogen, derivatization

## Abstract

2-Methoxyestradiol (2ME) is involved in the pathogenesis of preeclampsia and antitumor activity. In addition to its low concentration in healthy human serum, presence of isomers makes quantification of 2ME for clinical research and laboratory medicine difficult. The objective of this study was to develop a highly sensitive and accurate method for quantifying 2ME using LC-MS/MS combined with derivatization with 1-(2,4-dinitro-5-fluorophenyl)-4,4-dimethylpiperazinium iodide (MPDNP-F). This approach significantly enhanced the detectability of 2ME in positive electrospray ionization-tandem mass spectrometry (ESI-MS/MS) and enabled the chromatographic separation of 2ME from isomeric metabolites possessing a methoxy group, including 4-methoxyestradiol, 3-*O*-methyl 2-hydroxyestradiol, and 3-*O*-methyl 4-hydroxyestradiol (3M4OH). Although the derivatized 2ME and 3M4OH were closely eluted under the optimized LC conditions, the different fragmentation patterns of these isomers during MS/MS allowed their distinction. The lower limit of quantification for 2ME was 2.5 pg/mL, indicating a satisfactory sensitivity. These findings demonstrated that this LC-MS/MS method combined with the MPDNP-F derivatization can provide accurate and highly sensitive quantification of 2ME.

## Highlights

• MPDNP-F derivatization enabled highly sensitive assay of 2methoxy-estradiol (2ME).
• The sensitivity of quantitation of 2ME in serum was 2.5 pg/mL.
• Isomers interfering with 2ME could be separated, ensuring accurate quantitation.

## 1 Introduction

Estradiol (E2) is the most bioactive estrogen and has been widely measured in clinical tests and biological studies. E2 metabolism produces catechol estrogens, which are further metabolized by catechol-*O*-methyltransferase (COMT) to methoxy-estradiols (MEs). Recent studies show that 2-methoxyestradiol (2ME) is particularly elevated in late pregnancy, and low levels of 2ME during this period may be the main cause of preeclampsia [1,2]. Moreover, 2ME has been reported to exhibit antitumor activity [[3], [4], [5], [6], [7]]. Therefore, a highly sensitive and accurate quantification method for 2ME is required for clinical research and laboratory medicine in the analysis and treatment of 2ME-associated pathological conditions. However, the accurate analysis of 2ME in biological samples poses a great challenge due to its low concentration and the existence of its isomers. A separation method that can differentiate between 2ME and other MEs is necessary.

A few 2ME assays based on antigen-antibody reactions, such as radioimmunoassay [8] and enzyme-linked immunosorbent assay [9], have been reported. However, in some cases, immunoassays have insufficient sensitivity at low concentrations and insufficient specificity that may provide overestimated quantitative values [10].

Several highly sensitive analytical methods that combine liquid chromatography-tandem mass spectrometer LC-MS/MS and derivatization have been reported for differentiating among the pathophysiology of estrogen-associated diseases [[10], [11], [12], [13], [14], [15], [16]]. Regarding the specificity of LC-MS/MS analyses, production of fragment ions containing the estrogen skeleton is very effective in detection of estrogens in a complex matrix such as biological samples. Derivatization methods using pyridine-3-sulfonyl chloride [13], 1-(2,4-dinitro-5-fluorophenyl)-4-methylpiperazine (PDNP-F) [14,15], and 1-(2,4-dinitro- 5-fluorophenyl)-4,4-dimethylpiperazinium iodide (MPDNP-F) [16] are some of these methods with a high specificity.

Among them, derivatization using MPDNP-F provided a highly sensitive and specific quantification of E2 [16]. Although only E2 was quantified in the previous study [16], this derivatization procedure can determine other estrogen metabolites, including 2ME, with a high sensitivity and specificity.

Based on this background information, the objective of the present study was to develop a method for quantifying 2ME in human serum by LC-MS/MS and derivatization with MPDNP-F. To achieve the accurate quantification of 2ME, we scrutinized the separation of 2ME from other MEs, i.e., 4-methoxyestradiol (4ME), 3-*O*-methyl 2-hydroxyestradiol (3M2OH), and 3-*O*-methyl 4-hydroxyestradiol (3M4OH); the separation and occurrence of the 3-methylated metabolites (3M2OH and 3M4OH) have not been previously reported.

## 2 Materials and methods

### 2.1 Reagents and materials

Distilled water was obtained from Kanto Chemical Co. (Tokyo, Japan). Acetonitrile, methanol (LC-MS grade), formic acid, and 4-dimethylaminopyridine (DMAP) were purchased from Fujifilm Wako Pure Chemical Corporation (Osaka, Japan). MPDNP-F was synthesized in our laboratories as previously described [16].

MSG3000 (estrogen-free serum), 2ME, and 4ME were purchased from Sigma-Aldrich Japan (Tokyo, Japan). 2ME-^13^C_6_ (internal standard, IS), 3M4OH, and 3M2OH were obtained from Otsuka Pharmaceutical Co., Ltd. (Tokyo, Japan), Toronto Research Chemicals (Toronto, Canada), and BioDuro-Sundia (Shanghai, China), respectively. An Oasis® HLB μElution plate was purchased from Waters Corporation (Milford, MA, USA). Pooled human serum was purchased from Cosmo Bio Co. Ltd. (Tokyo, Japan).

### 2.2 Individual serum

Sera from healthy non-pregnant female individuals were purchased from Trina Bior Actives AG (Naenikon, Switzerland). Five donors (aged 18–37 years) were included. These samples were stored at −80 °C until use.

### 2.3 Standard solutions

Stock solutions of 2ME, 4ME, 3M2OH, 3M4OH, and 2ME-^13^C_6_ were prepared at a concentration of 1 ng/mL in acetonitrile. Calibration standards for 2ME were prepared at the concentrations of 5, 20, 50, 100, 150, and 200 pg/mL in MSG3000. The IS (2ME-^13^C_6_) solution was prepared in acetonitrile at a concentration of 50 pg/mL. These solutions were stored at −20 °C until use.

### 2.4 Quality control samples

Quality control samples (QC_LLOQ [lower limit of quantification], QCL [low, QCM [middle], and QCH [high]) were prepared by spiking 2ME standard in pooled serum (no 2ME was detected in the non-spiked pooled serum). The concentrations were 2.5, 10, 40, and 80 pg/mL, respectively.

### 2.5 Sample pretreatment

Extraction was performed using an Oasis HLB μelution plate. A calibration standard or serum sample (180 μL) was added to the IS solution (180 μL) and vortex-mixed for 1 min, followed by centrifugation (8000×*g*, 5 min, 25 °C. The supernatant was diluted with 390 μL of water. The extraction plate was serially equilibrated with acetonitrile, methanol, and water (200 μL each) prior to loading the sample. The sample was loaded onto the plate, washed with 200 μL of water and 200 μL of 50 % MeOH, and 2 ME and IS were eluted with 200 μL of acetonitrile. After evaporation of the solvent under a stream of nitrogen gas, the residue was added to 50 μL of MPDNP-F solution (0.4 mg/mL in acetonitrile) and 40 μL of DMAP solution (0.5 mg/mL in acetonitrile). The mixture was gently vortexed at 60 °Cfor 15 min. The derivatized sample was then dried under a stream of nitrogen gas. The residue was dissolved in 30 μL of 20 % acetonitrile solution.

### 2.6 LC-MS/MS analysis

LC-MS/MS analysis was performed using a QTRAP 4500 (AB SCIEX, Framingham, MA, USA) triple quadrupole mass spectrometer coupled to a 1290 Infinity I liquid chromatograph (Agilent Technologies, Santa Clara, CA, USA). LC separation was performed with an Ace Excell C18-PFP column (2.0 μm) 2.1 mm inner diameter × 100 mm (VWR, Radnor, PA, USA). The binary gradient system was used with 0.1 % (v/v) formic acid in water (mobile phase A) and 0.1 % (v/v) formic acid in acetonitrile (mobile phase B) at a flow rate of 0.4 mL/min. The column temperature was 35 °C. The analysis was performed in ESI (+) mode. The LC gradient condition and MS parameters are listed in Supplementary Table 1. This assay was used for 20 μL injections into the LC-MS/MS system. For increasing sensitivity, the capillary was narrowed to 0.065 μm (inner diameter) from the analytical column outlet to the MS detector. The Analyst 1.7 (SCIEX, Vaughan, ON, Canada) software and SCIEX OS were used for analysis and data processing. For 2ME quantification, peak area ratios (2ME-MPDNP/2ME-^13^C_6_-MPDNP, *y*) were plotted versus the concentrations of 2ME (pg/mL, *x*), and calibration curves were fitted with a weight factor of 1/*x*.

### 2.7 Evaluation of 2 ME assay

Optimal LC separations were determined using standard solutions of the MEs. For the 2ME quantification, we assessed intra- and inter-assay precision, LLOQ, stability, and accuracy. Intra- and inter-assay precisions were assessed by five repetitive measurements of four QC samples on one day and over five days, respectively. LLOQ was estimated by triplicate measurements of the diluted calibration standards with MSG3000. The criteria were determined based on the relative standard deviation (RSD, %) < 10, a signal-to-noise (S/N) ratio >20, and a deviation between theoretical and calculated values within 10 %. Accuracy was evaluated based on the recovery rates of the four QC samples.

Stability was assessed based on the average measured values of the QCL, QCM, and QCH samples (four replicates) before and after the freeze-thaw cycles at −80 °C (once and three times) and short-term storage at 4 °C (24 h and 72 h) and room temperature (24 h). Stability of derivatized 2ME was also assessed by reinjection of QCM and QCH samples after 24 h and 48 h, expressed as the average of % initial peak area/post-time peak area (N = 4).

To evaluate the applicability of this assay to a broad range of clinical uses, a 2ME serum sample at high concentration (10,000 pg/mL) was prepared by spiking pooled serum with a standard solution. A dilution test was performed to assess the assay's quantitative performance at high concentration levels. The high serum concentration was diluted 100-, 200-, and 400-fold with water and analyzed in triplicate.

## 3 Results

### 3.1 Mass spectra of derivatized MEs

**Fig. 1:**
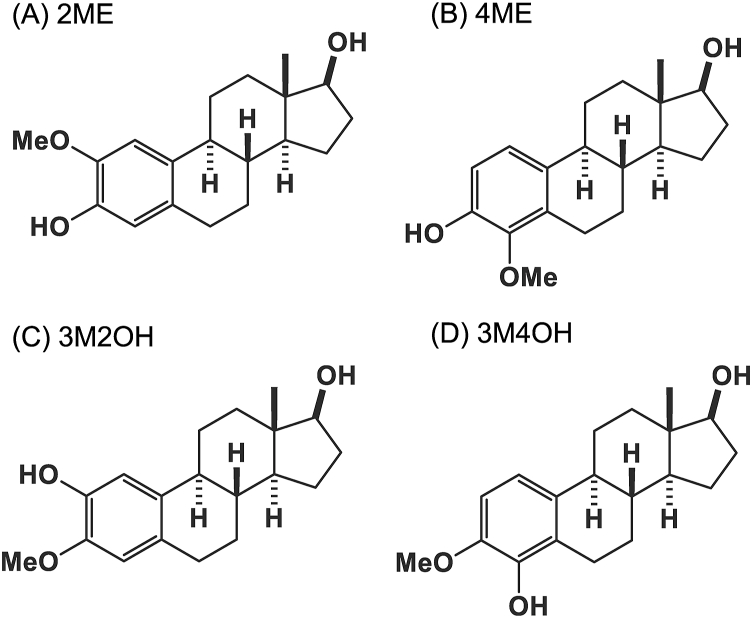
Chemical structures of MEs. 2-methoxyestradiol (2ME) A, 4-methoxyestradiol (4ME) 3 B, 3-O-methyl 2-hydroxyestradiol (3M2OH) C, and 3-O-methyl 4-hydroxyestradiol (3M4OH) D.

**Fig. 2:**
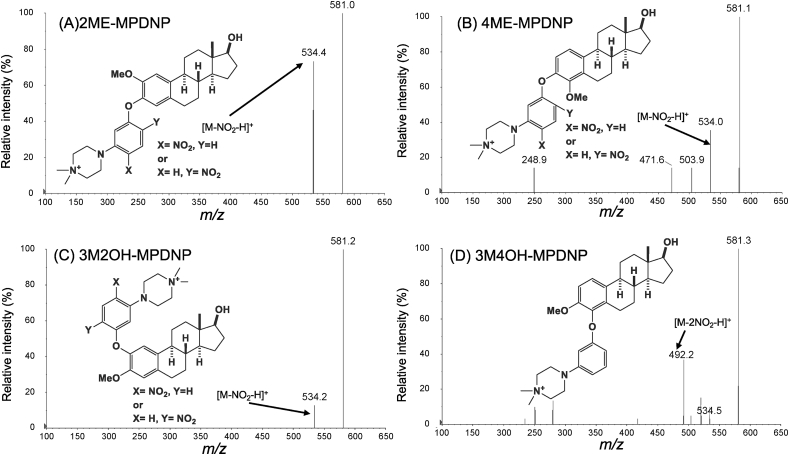
Product ion spectra of derivatized MEs. 2ME (A), 4ME (B), 3M2OH (C), and 3M4OH (D).

The structures of the MEs examined in this study are shown in Fig. 1. These MEs were derivatized with MPDNP-F. MEs were successfully derivatized under the same conditions as previously reported [16]. All the derivatives of these MEs provided intense [M]^+^. Their fragment ions were acquired by product ion scan (Fig. 2). [M-NO_2_-H]^+^ were detected as the major fragment ions at *m*/*z* 534 for the derivatized MEs as with the case of E2 [16]. These ions contained the estrogen skeleton, which enabled the specific detection of the MEs. Only the derivatized 3M4OH gave [M-2NO_2_-H]^+^ at *m*/*z* 492. Based on these results, selected reaction monitoring transitions for detection of the derivatized estrogens were set as follows: 2ME, 4ME, 3M2OH: 581.1 > 534.4, and 3M4OH: 581.1 > 492.0, respectively (Supplementary Table 1).

### 3.2 Chromatographic separation of MEs

**Fig. 3:**
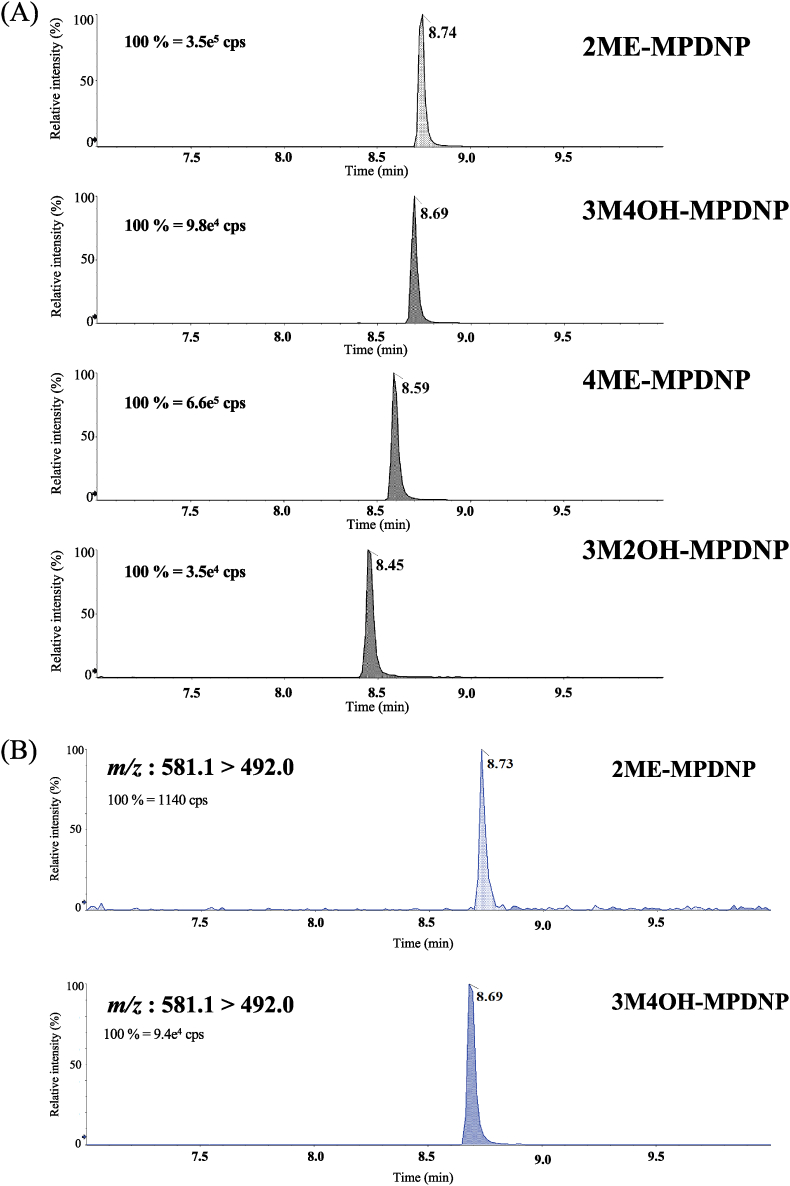
Chromatographic separation of MEs. (A) SRM chromatograms of derivatized MEs with transition for 2ME quantification (*m/z*: 581.1 > 534.4). (B) SRM chromatograms of 2ME-MPDNP and 3M4OH-MPDNP with transition *m/z*: 581.1 > 492.0.

The chromatographic separation of the MEs is shown in Fig. 3. The derivatized 2ME, 4ME, 3M2OH, and 3M4OH were detected at 8.74, 8.59, 8.45, and 8.69 min, respectively. The total run time was 13 min. The resolution (*Rs*) and separation factor (α) were calculated based on the following formulas: *Rs* = 1.18 × (*t*_R2_ – *t*_R1_)/(*W*_h1_ + *W*_h2_), where *t*_R_ is the retention time and *W*_h_ is the half width of the peak.α = (*t*_R2_ – *t*_0_)/(*t*_R1_ – *t*_0_), where *t*_0_ (holdup time) = 0.57 min. Under the determined LC conditions, the *Rs* and α values for 2ME-MPDNP and 3M2OH-MPDNP were 4.72 and 1.04, and 2ME-MPDNP and 4ME-MPDNP were 2.58 and 1.02, respectively. These isomers were completely separated from 2ME-MPDNP. In contrast, the *Rs* and α values for 2ME-MPDNP and 3M4OH-MPDNP were 0.87 and 1.00, respectively; thus, the chromatographic separation was insufficient. However, 3M4OH-MPDNP gave a specific product ion [M-2NO_2_-H]^+^ at *m/z* 492, and the selected reaction monitoring transitions 581.1 > 492.0 could be used to confirm whether 3M4OH was present in the sample. MPDNP-F derivatization enables isomer differentiation even when LC separation is insufficient.

### 3.3 Performance of 2ME assay

Assay performances are shown in Table 1. The reproducibility and linearity of the calibration curves were evaluated by preparing five curves. The regression formula was *y* = (0.06592 ± 0.00302)*x* + (0.01030 ± 0.03622) (mean ± SD were given for the slopes and y-intercepts). All the curves showed a linearity in the range of 5.0–200 pg/mL with a determination coefficient of (*r*^2^) > 0.997. The RSDs of intra-assay and inter-assay measurements (N = 5) were <10.0 % for the four QC samples. The recoveries (%) of four QC samples were within 93–113 %. The LLOQ of 2ME was 2.5 pg/mL. Thus, the developed method had not only good precision and accuracy, but also twice as high sensitivity as the previously reported method [15] for the 2ME quantification. 2 ME in serum was stable for up to three freeze-thaw cycles at −80 °C Short-term storage was stable at 4 °C for 24-h and 72-h, while variation in quantitative values (%) of three QC samples was <10.0 %. The stability at 25 °C for 24-h variation in quantitative values was worse than at 4 °C. It should be stored at a low temperature (<4 °C) prior to measurement in human serum.

**Table 1:** Assay precision and accuracy.

Level	Nominal concentration (pg/mL)	Intra-assay precision average (pg/mL)	RSD (%)	Intra-assay recovery (%)	Inter-assay precision average (pg/mL)	RSD (%)	Inter-assay recovery (%)
QC_LLOQ	2.5	2.37	5.9	94.8	2.33	6.2	93.2
QCL	10	10.8	9.9	108.0	11.2	6.9	112.1
QCM	40	41.4	5.3	103.5	40.6	3.1	101.5
QCH	80	76.2	3.1	95.3	80.6	3.9	100.7

RSD, relative standard deviation; QC_LLOQ, quality control lower limit of quantification; QCL, quality control low; QCM, quality control middle; QCH, quality control high.

The stability of derivatized 2ME was assessed by reinjection of QCM and QCH. The peak area decrease of each QC sample was less than 15 % after 48 h. The samples were stable in the autosampler (10 °C) for at least 24 h (Table 2). The results of the dilution test are presented in Supplementary Table 2. The recovery rates of the diluted serum ranged between 82 % and 95 %. These results demonstrated that high-concentration 2ME serum could be accurately quantified through dilution.

**Table 2:** Stability in 2ME quantification.

Freeze-thaw stability (N = 4)		QCL	QCM	QCH
Temperature	Cycle	Concentration (%)		
−80 °C	0	100	100	100
	cycle 1	101.9	101.2	99.3
	cycle 3	96.2	95.8	100.7

Short term storage (N = 4)		QCL	QCM	QCH
Temperature	Time	Concentration (%)		
4 °C	0	100	100	100
	24 h	91.8	92.1	92.9
	72 h	98.9	101.2	97.7
25 °C	24 h	87.3	91.9	94.2

Stability after derivatization (N = 4)		QCM	QCH
Temperature	Time	Peak area (%)	
10 °C	0	100	100
	24 h	109.0	102.3
	48 h	89.5	98.6

2 ME, 2-methoxyestradiol; QCL, quality control low; QCM, quality control middle; QCH, quality control high.

### 3.4 Quantification of serum 2ME

**Fig. 4:**
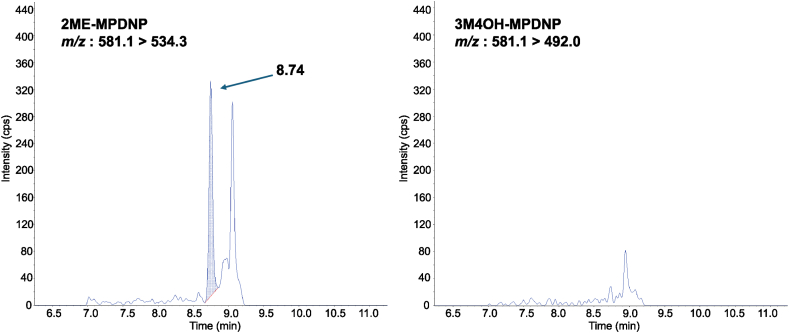
SRM chromatogram of 2ME-MPDNP in human serum. SRM chromatogram of sample Number 2 was shown. The measured concentration of 2ME was 5.25 pg/mL.

The developed method was used to analyze the serum from six healthy non-pregnant female individuals to evaluate the applicability of the method. Representative selected reaction monitoring chromatograms of 2ME, 4ME, 3M2OH: 581.1 > 534.4, and 3M4OH: 581.1 > 492.0 in serum and are shown in Fig. 4, which demonstrated that 2ME can be separated from some interfering peaks and detected with a low background noise. In this study, selected reaction monitoring chromatograms of 581.1 > 492.0 (Fig. 4) revealed that 3M4OH was practically absent in healthy non-pregnant female human serum. Although the LC separation of 2 ME and 3M4OH is insufficient in chromatograms of 581.1 > 534.4, the specificity of this method is sufficient as the absence of 3M4OH was demonstrated. 2ME was successfully quantified in three of the six samples (Table 3), and 3M4OH, 3M2OH, 4ME were not detected in the samples in which 2 ME could be quantified. No MEs including 2ME were detected in three samples.

**Table 3:** Serum 2 ME concentrations of healthy non-pregnant women.

Sample number	Age	2 ME (pg/mL)
1	18	< LLOQ
2	27	5.25
3	30	6.44
4	35	< LLOQ
5	36	< LLOQ
6	37	4.95

2 ME, 2-methoxyestradiol.

## 4 Discussion

We established a method for quantifying 2ME in serum using MPDNP-F derivatization and LC-MS/MS. This assay enabled specific quantification of 2ME as distinguished from 4ME, 3M2OH, and 3M4OH, which are produced in the COMT-mediated catechol estrogen metabolic pathway [1,[3], [4], [5]]. Previous studies indicated that the methyl transfer reaction differs between the hydroxy groups at the 3 and 2 positions of the steroid A ring [17,18]; however, there are no detailed reports investigating 3M2OH and 3M4OH, apart from studies on 2ME and 4ME. To the best of our knowledge, our study is the first reported instance to quantify 2ME in serum when the four isomeric MEs can be separated by LC-MS/MS. The separation of four isomers involved in the metabolic pathway of catechol estrogens enabled a comprehensive analysis of MEs, including the isomers methylated at the 3 positions (3M2OH and 3M4OH), which have been poorly studied. Moreover, a highly sensitive analysis of 2ME with a small serum volume (180 μL) achieved LLOQ: 2.5 pg/mL. Quantitative sensitivity was improved 2-fold in comparison to previously reported measurements (LLOQ: 5 pg/mL) using a high-end instrument (SCIEX QTrap 6500^+^) and 500 μL of serum [15]. Serum 2ME concentrations in non-pregnant women have been reported to be very low, with blood levels ranging from 18 to 138 pg/mL as measured by ELISA [9]. In this study, 2ME was quantifiable in only 3 out of 6 serum samples from healthy non-pregnant women. The low detection rate observed, despite the high sensitivity of the LC-MS/MS assay, may have been attributed to differences in assay methodologies. Previously reported LC-MS/MS assays in healthy women failed to detect 2ME, despite a quantitative sensitivity of 5 pg/mL [15]. In contrast, the higher detection rates reported with ELISA might reflect its limited specificity and quantification accuracy, potentially leading to overestimation. Actual 2ME blood levels in non-pregnant women might even be lower than those previously reported. This should be confirmed in studies with larger sample sizes involving measurement using the newly developed LC-MS/MS method.

We could completely separate 4ME and 3M2OH. Moreover, 3M4OH was not detected in healthy non-pregnant women. Therefore, this method was suitable for quantification of 2ME in human serum. Notably, 2ME has been studied due to its possible involvement in preeclampsia and antitumor activity [[2], [3], [4], [5], [6], [7]] as well as its neurotoxic properties with significantly higher concentrations in patients with Parkinson's disease [19]. For exploring the relevance of 2ME in disease, future studies should investigate how 2ME and other MEs fluctuate in various pathological conditions.

As our study had a small sample size, a large-scale study will be required to verify the clinical value of the method. Nevertheless, this method showed a great potential for application in laboratory medicine as a highly sensitive technique for the quantification of 2ME.

## 5 Conclusion

We developed a method using LC-MS/MS combined with MPDNP-F derivatization for quantification of 2ME. This assay enables highly sensitive and accurate quantification of 2ME with a small volume of serum (180 μL). Although this is a preliminary study, we demonstrated that this method is applicable in clinical laboratory medicine.

## CRediT authorship contribution statement

**Koji Takahashi:** Writing – original draft, Methodology, Investigation, Formal analysis, Data curation, Conceptualization. **Masaki Takiwaki:** Writing – original draft, Methodology, Investigation, Conceptualization. **Seketsu Fukuzawa:** Writing – review & editing, Methodology, Investigation. **Yoshikuni Kikutani:** Writing – review & editing, Methodology, Investigation. **Kentaro Abe:** Writing – review & editing, Methodology, Investigation. **Tatsuya Higashi:** Writing – review & editing, Supervision, Resources. **Hironori Kobayashi:** Writing – review & editing, Supervision, Resources.

## Data statement

Data will be made available on request.

## Funding sources

This research did not receive any specific grant from funding agencies in the public, commercial, or not-for-profit sectors.

## Declaration of competing interest

The authors declare that they have no known competing financial interests or personal relationships that could have appeared to influence the work reported in this paper.

## Data Availability

Data will be made available on request.
