# Supplementary material for: Development of an accurate and sensitive assay for 2-methoxyestradiol using derivatization and liquid chromatography-tandem mass spectrometry
Source: Pract Lab Med. 2025 Jan 9;44:e00447. doi: 10.1016/j.plabm.2024.e00447 (PMC11772991; doi:10.1016/j.plabm.2024.e00447)
Supplement: Multimedia component 1 [file mmc1.docx]

**Supplementary Table 1. Assay conditions for LC-MS/MS systems.** Declustering Potential (DP), and Collision Energy (CE).

| **LC conditions** | | | | |
| --- | --- | --- | --- | --- |
|  | Time  (min) | Flow Rate  (mL/min) | Mobile phase  A (％) | Mobile phase  B (％) |
| Gradient profile | 0 | 0.4 | 80 | 20 |
|  | 0.5 | 0.4 | 68 | 32 |
|  | 7 | 0.4 | 59 | 41 |
|  | 9 | 0.4 | 10 | 90 |
|  | 11 | 0.4 | 10 | 90 |
|  | 11.01 | 0.4 | 80 | 20 |
|  | 13 | 0.4 | 80 | 20 |
| **MS conditions** | | | | |
| Compound (Derivatized with MPDNP) | Precursor ion  *(m/z)* | Product ion  (*m/z)* | DP  (V) | CE  (V) |
| 2ME | 581.1 | 534.4 | 160 | 64 |
| 4ME | 581.1 | 534.4 | 160 | 64 |
| 3M2OH | 581.1 | 534.4 | 160 | 64 |
| 3M4OH | 581.1 | 492.0 | 160 | 64 |
| 2ME-^13^C_6_ | 587.1 | 540.4 | 160 | 64 |

**Supplementary Table 2. Dilution test of high concentration 2ME serum.**

| Dilution rate (fold) | Nominal concentration  (pg/mL) | Calculated average concentration (pg/mL) | RSD (%) | Recovery (%) |
| --- | --- | --- | --- | --- |
| 100 | 100 | 94.9 | 10.4 | 94.9 |
| 200 | 50 | 41.2 | 10.0 | 82.4 |
| 400 | 25 | 21.1 | 2.7 | 84.4 |

RSD, relative standard deviation.
